# Protocol for optimized mononuclear cell isolation from liver and tumor tissue using mechanical or enzymatic digestion

**DOI:** 10.1016/j.xpro.2025.104289

**Published:** 2025-12-19

**Authors:** Sachin Kumar Singh Chauhan, Linda Feldbrügge, Moritz Schmelzle, Heiner Wedemeyer, Bernd Heinrich

**Affiliations:** 1 Department of Gastroenterology, Hepatology, Infectious Diseases and Endocrinology, Hannover Medical School, 30625 Hannover, Lower Saxony, Germany; 2 Department of General, Visceral and Transplant Surgery, Hannover Medical School, 30625 Hannover, Lower Saxony, Germany

**Keywords:** cancer, cell isolation, flow cytometry, Immunology, single cell

## Abstract

Mononuclear cells from human liver tissue, including tumor and non-tumor regions, are a valuable source for studies of the tumor microenvironment. Here, we present a protocol to dissociate liver tissue from patients with hepatocellular carcinoma. We provide steps to generate single-cell suspension using either mechanical or enzymatic digestion, following steps for separating mononuclear cells using density gradient. Finally, we describe steps for flow cytometry staining of isolated immune cells.

For complete details on the use and execution of this protocol, please refer to Moreno-Fernandez et al.^1^ and Heinrich et al.^2^

## Highlights

• Steps from resected tissue to single-cell suspension of immune cells
• Instructions for efficient digestion and isolation with reduced experimenter bias
• Evaluation of both techniques using qualitative and quantitative measurements
• Guidance on analyses of low-abundance immune cells

## Abstract (editor highlights)

Publisher’s note: Undertaking any experimental protocol requires adherence to local institutional guidelines for laboratory safety and ethics.

## Graphical abstract

**Figure:**
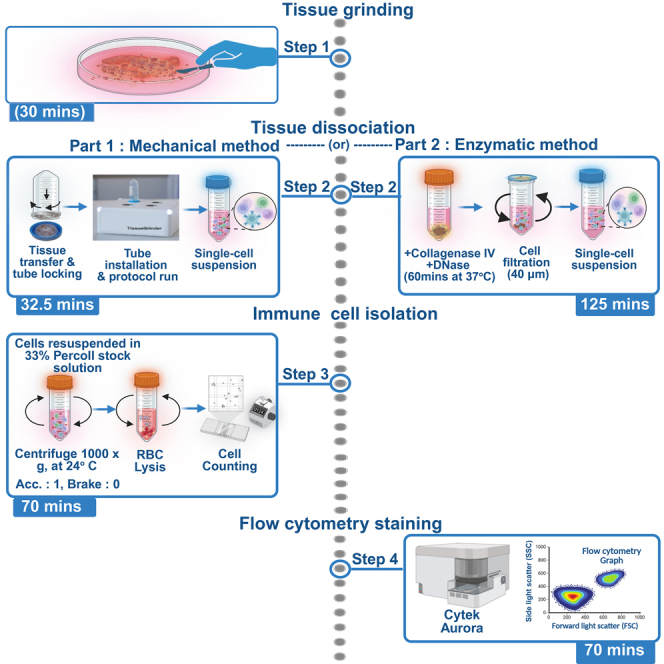

## Before you begin

The liver provides a reservoir of diverse immune cells that support essential physiological processes but may also contribute to oncogenesis. Hepatocellular carcinoma (HCC) is the most common liver tumor. Approximately 35% of HCC tumors are classified as ‘inflamed,’ which show strong immune cell infiltration.^3^^,^^4^^,^^5^ Resection of the HCC tumor is a preferred treatment for early-stage HCC. Resected tissue is a valuable source to study immune cells.^6^^,^^7^ Here, we discuss two protocols to perform tissue digestion and prepare single-cell suspensions to isolate viable immune cells for tumor immunology studies. The following protocols generate single-cell suspensions using either mechanical digestion with TissueGrinder or enzymatic digestion with Collagenase IV and DNase, adapted from previously published protocols.^1^^,^^2^ Potential challenges include low cell yield due to fibrotic or cirrhotic liver tissue, reduced viability due to stress during tissue processing, and necrotic or fatty tissue composition also resulting in poor immune cell quality and yield.

### Innovation statement

This article presents two optimized protocols for isolating mononuclear cells (MNCs) from liver tissue: a rapid mechanical method and an enzymatic method.

Begin with the mechanical protocol to isolate MNCs from both tumor and non-tumor regions of liver tissue resected from HCC patients. Replace traditional digestion with Collagenase IV and DNase I by applying TissueGrinder technology. Perform mechanical dissociation to accelerate tissue processing, cut costs by eliminating expensive enzymes, and reduce variability introduced by enzymatic incubation. Preserve cell viability, protect surface marker integrity, and broaden the diversity of isolated cells. Capture rare immune subsets such as ILCs with higher efficiency. Rely on this method as a fast, cost-effective, and reproducible alternative to enzyme-based digestion that strengthens immune profiling in liver immunology research.

In case of enzymatic digestion is selected, follow the second optimized protocol. Apply a single, fixed concentration of density gradient medium rather than performing complex layering steps. Streamline preparation, improve reproducibility, and avoid errors such as inaccurate layering or unwanted mixing. Maintain high viability of isolated cells while ensuring consistency across experiments.

Use both protocols to address the challenge of dissociating cirrhotic liver tissue, which often resists standard methods. Generate highly viable single-cell suspensions suitable for immediate downstream applications. Proceed directly to flow cytometry or single-cell sequencing without additional handling.

Adopt these approaches to maximize efficiency, lower costs, and guarantee reproducibility in MNC isolation. Enable accurate profiling of immune subsets and accelerate insights into tumor and non-tumor immune responses in HCC.

### Institutional permissions

Informed consent was obtained from patients providing samples, and all experiments were conducted in accordance with the Declaration of Helsinki. The protocol for obtaining human biopsy samples is approved by the ethics committee of Hannover Medical School (No. 10855_BO_K_2023).

### Preparation of the environment and materials

**Timing: Flexible before experiment**

Decontaminate the working space and equipment inside the biosafety cabinet using 70% ethanol to maintain sterility and prevent contamination. Pre-cool the centrifuge to 4°C and preheat the water bath to 37°C.

1. Tissue storage preparation.
  a. Transfer cold MACS tissue storage solution into a 50 mL tube, ensuring the amount used can fully submerge the tissue sample. Immediately transfer the tissue into the solution post-surgery.
    **Storage conditions:** Store and maintain MACS tissue storage solution at 4°C until product expiry.
    ***Note:*** Ensure the tissue stays fully submerged to prevent dryness and necrosis.
    ***Note:*** If tissue delivery is delayed and same-day tissue processing is not feasible, place the tissue in the tissue storage solution and store it at 4°C for up to 12–14 h. Process the tissue the following day.
  b. Weigh the tissue first and NOTE down the weight of non-tumoral and tumor tissue section in the biosafety cabinet.
2. Prepare Percoll stock solution.
  a. Prepare a 33% Percoll gradient medium by mixing 33% (v/v) Percoll solution with 67% (v/v) RPMI medium (without FCS). Mix thoroughly to ensure homogeneity and keep the solution at 20°C–25°C until use.

**Storage conditions:** Prepare the Percoll stock solution and store it at 20°C–25°C for same-day use only.

***Note:*** Prepare 30 mL of 33% Percoll solution for each gram of tissue. Begin by weighing the tissue to determine the necessary volume of 33% Percoll solution.

***Note:*** Prepare the 33% Percoll solution fresh on the day of isolation and keep it at 20°C–25°C until use.

3. Thawing and transferring cell culture reagents.
  a. Thaw DNase I, Collagenase IV, to reach 20°C–25°C (Only Protocol 2).
  b. Thaw Gibco Recovery Cell Culture Freezing Medium.
    **Storage conditions**: Store and maintain it at 4°C until 24 hours. Protect from light.
  c. Transfer Percoll solution, RPMI (without FCS), RPMI + 10% FCS, PBS and RBC lysis buffer aliquots to 20°C–25°C (Storage conditions).
    **CRITICAL:** All time-consuming steps, such as preparing Percoll solutions thawing enzymes and media, setting PBS and reagents to 20°C–25°C or 4°C and pre-cooling the centrifuge should be completed in advance whenever possible. Preparing these steps before surgical sampling is essential, as the quality of results relies heavily on the rapid processing of resected liver cells post resection.

### Preparation of instruments

**Timing: Flexible before experiment**

Before beginning the experiment, ensure all instruments are powered on, initialized, and set to the correct operating conditions. The following steps outline the required preparation to confirm the TissueGrinder system and incubator are ready for use.

4. Power on the TissueGrinder Instrument.
  a. Switch on instrument which indicated by white color light around all four corners.
  b. Turn-on the computer and login into the TissueGrinder software.
5. Switch on the swinging rotor incubator and set it to 37°C.

## Key resources table

**Table:** 

REAGENT or RESOURCE	SOURCE	IDENTIFIER
**Antibodies**		

Anti-human CD45 Antibody - APC/Fire 810 (Clone: HI30); Antibody working dilution: 1:100	BioLegend Europe B.V., the Netherlands	Cat# 304076, RRID:AB_2860792
Anti-human CD34 Antibody – FITC (Clone: 581); Antibody working dilution: 1:30	BioLegend Europe B.V., the Netherlands	Cat# 343504, RRID:AB_1731852
Anti-human CD123 Antibody – FITC (Clone: 6H6); Antibody working dilution: 1:30	BioLegend Europe B.V., the Netherlands	Cat# 306014, RRID:AB_2124259
Anti-human CD14 Antibody – FITC (Clone: M5E2); Antibody working dilution: 1:30	BioLegend Europe B.V., the Netherlands	Cat# 301804, RRID:AB_314186
Anti-human CD19 Antibody – FITC (Clone: HIB19); Antibody working dilution: 1:30	BioLegend Europe B.V., the Netherlands	Cat# 302226, RRID:AB_493751
Anti-human CD11c Antibody – FITC (Clone: Bu15); Antibody working dilution: 1:30	BioLegend Europe B.V., the Netherlands	Cat# 337214, RRID:AB_2129792
Anti-human FcεRIα Antibody – FITC (Clone: AER-37/CRA-1); Antibody working dilution: 1:30	BioLegend Europe B.V., the Netherlands	Cat# 334608, RRID:AB_1227653
Anti-human TCR α/β Antibody – FITC (Clone: IP26); Antibody working dilution: 1:30	BioLegend Europe B.V., the Netherlands	Cat# 306706, RRID:AB_314644
Anti-human TCR γ/δ Antibody – FITC (Clone: B1); Antibody working dilution: 1:30	BioLegend Europe B.V., the Netherlands	Cat # 331208, RRID:AB_1575108
Anti-human CD3 Antibody - Brilliant Violet 570 (Clone: UCHT1); Antibody working dilution: 1:30	BioLegend Europe B.V., the Netherlands	Cat# 300436, RRID:AB_2562124
Anti-human CD4 Antibody - Brilliant Violet 650 (Clone: RPA-T4); Antibody working dilution: 1:30	BioLegend Europe B.V., the Netherlands	Cat# 300536, RRID:AB_2632791
Anti-human CD8a Antibody - Brilliant Violet 510 (Clone: RPA-T8); Antibody working dilution: 1:30	BioLegend Europe B.V., the Netherlands	Cat# 301048, RRID:AB_2561942
Anti-human CD16 Antibody - Alexa Fluor 700 (Clone: 3G8); Antibody working dilution: 1:30	BioLegend Europe B.V., The Netherlands	Cat# 302026, RRID:AB_2278418
Anti-human CD56 Antibody - Brilliant Violet 605 (Clone: HCD56); Antibody working dilution: 1:30	BioLegend Europe B.V., the Netherlands	Cat# 318334, RRID:AB_2561912
Anti-human CD94 Antibody - PE/Dazzle594 (Clone: DX22); Antibody working dilution: 1:30	BioLegend Europe B.V., the Netherlands	Cat# 305520, RRID:AB_2734277
Anti-human CD161 Antibody - Brilliant Violet 785 (Clone: HP-3G10); Antibody working dilution: 1:30	BioLegend Europe B.V., the Netherlands	Cat# 339930, RRID:AB_2563968
Anti-human CD127 (IL-7Rα) Antibody - Brilliant Violet 711 (Clone: A019D5); Antibody working dilution: 1:30	BioLegend Europe B.V., the Netherlands	Cat# 351328, RRID:AB_2562908

**Biological samples**		

Surgically removed tissue of hepatocellular carcinoma tumor and sample originating from tumor-surrounding, non-tumorous liver tissue.	Hannover Medical School, Hannover, Germany	N/A

**Chemicals, peptides, and recombinant proteins**		

RPMI Medium 1640 (1×) + GlutaMAX-I	Gibco/Thermo Fisher Scientific FEI Deutschland GmbH, Germany	Cat# 61870-010
Fetal Bovine serum (FBS), Qualified	Gibco/Thermo Fisher Scientific FEI Deutschland GmbH, Germany	Cat# 10270-106
MACS tissue storage solution	Miltenyi Biotec B.V. & Co. KG, Germany	Cat# 130-100-008
Collagenase type IV (1 mg/mL)	STEMCELL Technologies Germany GmbH, Germany	Cat# 07909
Deoxyribonuclease I (DNase I solution [1 mg/mL])	STEMCELL Technologies Germany GmbH, Germany	Cat# 07900
Percoll	Cytiva life sciences/Fisher Scientific GmbH, Germany	Cat# 10607095
1× RBC lysis buffer	eBiosciences/ThermoFisher Scientific, Germany	Cat# 00-4333-57
Dulbecco’s Phosphate Buffered Solution (DPBS (1×))	Gibco/Thermo Fisher Scientific FEI Deutschland GmbH, Germany	REF# 14190-094
Human TruStain FcX (Fc receptor blocking solution); Working dilution: 1:100 in PBS	BioLegend Europe B.V., the Netherlands	Cat# 422302
Zombie NIR Fixable Viability Kit; Working dilution: 1:100 in FACS buffer	BioLegend Europe B.V., the Netherlands	Cat# 423106

**Software and algorithms**		

FlowJo_v.10.10.0	Becton Dickinson, Belgium	https://www.flowjo.com/
Prism- GraphPad 10.1.2	GraphPad Software, United States	https://www.graphpad.com/

**Other**		

Student Anatomical Standard Forceps, Length: 11.5 cm, Net Weight: 16 g, Tip Shape: Straight, Tip Width: 2.5 mm, Tips: Serrated	Fine Science Tools GmbH, Heidelberg, Germany	Cat# 91100-12
Student Anatomical Standard Forceps, Length: 13 cm, Net Weight: 19.5 g, Tip Shape: Straight, Tip Width: 2.7 mm, Tips: Serrated	Fine Science Tools GmbH, Heidelberg, Germany	Cat# 91100-13
Student Anatomical Standard Forceps, Length: 14.5 cm, Net Weight: 24 g, Tip Shape: Straight, Tip Width: 3 mm, Tips: Serrated	Fine Science Tools GmbH, Heidelberg, Germany	Cat# 91100-14
Student Anatomical Standard Forceps, Length: 16 cm, Net Weight: 31 g, Tip Shape: Straight, Tip Width: 3.5 mm, Tips: Serrated	Fine Science Tools GmbH, Heidelberg, Germany	Cat# 91100-16
Student Dumont #7 Forceps, Length: 11 cm, Net Weight: 14 g, Style: #7, Tip Dimensions: 0.17 × 0.1 mm, Tip Shape: Curved, Tips: Standard	Fine Science Tools GmbH, Heidelberg, Germany	Cat# 91197-00
Student Anatomical Standard Forceps, Length: 17 cm, Net Weight: 29.5 g, Tip Shape: Straight, Tip Width: 4 mm, Tips: Serrated	Fine Science Tools GmbH, Heidelberg, Germany	Cat# 91100-17
Tube 50 mL, 114 × 28 mm, PP	Sarstedt AG & Co. KG, Germany	REF# 62.547.254
Tube 15 mL, 120 × 17 mm, PP	Sarstedt AG & Co. KG, Germany	REF# 62.554.502
Tissue culture dish 100, Standard	Sarstedt AG & Co. KG, Germany	REF# 83.3902
Disposable scalpel No. 21 pfm (Stainless steel blade with plastic handle)	Feather Safety Razor Co., Ltd., Osaka, Japan	Cat# 02.001.30.021
Cell strainer 40 μm Nylon	Falcon/Corning GmbH, Germany	REF# 352340
Filtropur S, 0.2 μm	Sarstedt AG & Co. KG, Germany	REF# 83.1826.001
Tube 5 mL, 75 × 12 mm, PS (FACS staining tubes)	Sarstedt AG & Co. KG, Germany	REF# 55.1579
LUNA 2-channel cell counting slides	BioCat GmbH, Germany	Cat# L12001-LG
Luna FL Automated Fluorescence cell counter	Logos Biosystems, France	SKU: L20001
Incubator hood TH 30 (Temperature-controlled Orbital shaker)	Edmund Bühler GmbH, Germany	Order No# 6162 000
Megafuge ST Plus Series Centrifuge (Temperature-controlled desktop centrifuge)	Thermo Fisher Scientific FEI Deutschland GmbH, Germany	https://www.thermofisher.com/de/de/home.html
Spectral flow cytometer	Cytek Biosciences B.V, the Netherlands	Cytek Aurora Cytometer
FFX-TissueGrinder Benchtop Unit with 4 slots	Fast Forward Discoveries GmbH, Germany	ID# TG-BT-4
FFX Tissue Grinder Milling Set (TG tube) (single use including 50 mL Falcon tubes and cell sieves 70 μm, sterile)	Fast Forward Discoveries GmbH, Germany	ID # TG-C-70-S
Weighing Modules	Sartorius GmbH, Germany	Cubis High capacity Balance
Eye-protection safety glasses	3M Science Applied to Life, Germany	https://www.3mdeutschland.de/3M/de_DE/p/c/personliche-schutzausrustung/augenschutz/sicherheitsbrillen/

## Materials and equipment

**Table:** Complete RPMI-1640 (C-RPMI)

Reagent	Final concentration	Amount
RPMI-1640 medium	90%	450 mL
FBS	10%	50mL

**Storage conditions**: C-RPMI can be prepared in advance and stored at 4°C for several weeks before use.

***Note:*** It is known that antibiotics in cell culture media can alter gene expression and regulation.^8^ To prevent such effects, we omitted antibiotics from the culture medium for this short-term protocol, not requiring extended cell culture.

**Table:** Flow cytometry staining buffer (FACS Buffer)

Reagent	Final concentration	Amount
1× DPBS	99.6%	49.8 mL
BSA	1%	0.5 g
EDTA	2mM	200 μL of 0.5M EDTA solution
Sodium Azide	0.1%	0.05 g

**Storage conditions:** Prepare the FACS buffer, filter it using a 0.2 μm cell strainer, store it at 4°C, and use it within two weeks.

### Preparation of Zombie NIR fixable viability kit

Prepare the working solution of the Zombie NIR Fixable Viability Kit, which contains lyophilized Zombie NIR™ dye and anhydrous DMSO. Bring the kit to 20°C–25°C, then reconstitute the dye by adding 100 μl of DMSO to one vial of Zombie NIR™ dye. Mix until the dye dissolves completely.

**Storage conditions:** Store the kit at −20°C upon receipt. Keep vials sealed until use. After adding DMSO to the Zombie NIR™ dye, use the solution immediately or store it at −20°C in a dry, light-protected environment. Place it in a desiccator or a container with desiccant, and use it within one month.

## Step-by-step method details

### Tissue grinding

**Timing: 30 min for 1 g of tissue**

For mechanical digestion, refer to steps 1–4 and 5a; for enzymatic digestion, refer to steps 1–4 and 5b. This section describes the preparation of liver tumor and non-tumor tissue before dissociation. Keep the tissue chilled in MACS storage solution, weigh it, and mince it into 0.5–1 mm fragments while ensuring it remains fully submerged to prevent drying. Transfer the minced material into labeled tubes and wash with DPBS if excess blood is present. Adjust the volume with RPMI-1640, centrifuge, and discard the supernatant. Resuspend the pellet in RPMI-1640 for either mechanical or enzymatic digestion, calculating volumes according to tissue weight.

**CRITICAL:** Wear appropriate personal protective equipment based on the potential hazards of the tissue and in accordance with local safety regulations.

1. Weigh the non-tumor and tumor sections in a petri dish submerged in cold tissue storage solution and record their weights.

***Note:*** During processing, keep the liver tissue in the laminar flow hood ensuring it stays submerged in cold MACS tissue storage solution.

***Note:*** Ensure the tissue remains submerged in MACS tissue storage solution and remain mostly on ice, as tissue drying can cause necrosis and result in low viable cell yield.

2. Mince tissue sections into 0.5–1 mm pieces using a number 21 pfm safety scalpel while holding the tissue with forceps in a petri dish containing MACS tissue storage solution.

**Figure 1:**
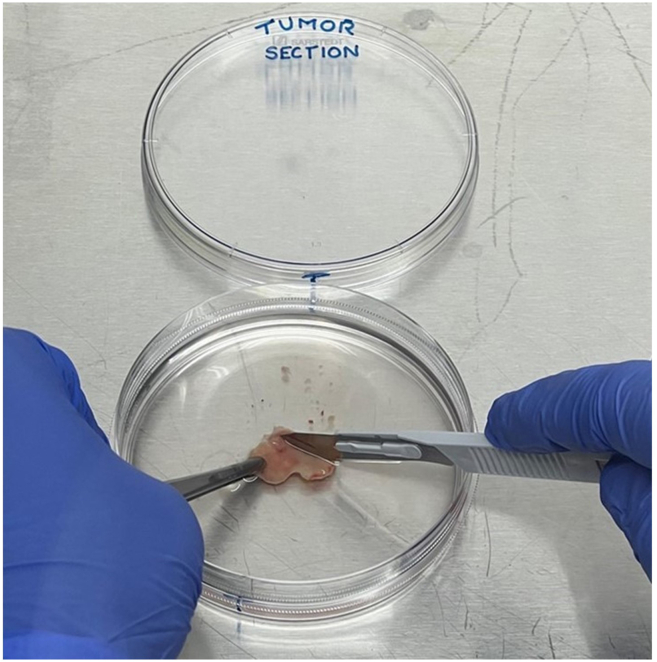
Illustration of a tissue biopsy submerged in a tissue storage solution, demonstrating the proper technique for submerging, holding, and mincing the tissue

***Note:*** If the solution does not fully submerge the tissue pieces, add cold RPMI-1640 media (without FCS) to maintain submersion (Figure 1).

3. Pipette the ground tissue suspension, which contains a mixture of MACS tissue storage solution and RPMI-1640 medium, and transfer it into a labeled 50 mL tube designated as non-tumor or tumor section.

***Note:*** If the tissue sample contains excess blood, wash it again with 1× DPBS by centrifugation at 500 × *g* for 10 min at 4°C to further reduce blood contamination, which can interfere with downstream processing.

**Figure 2:**
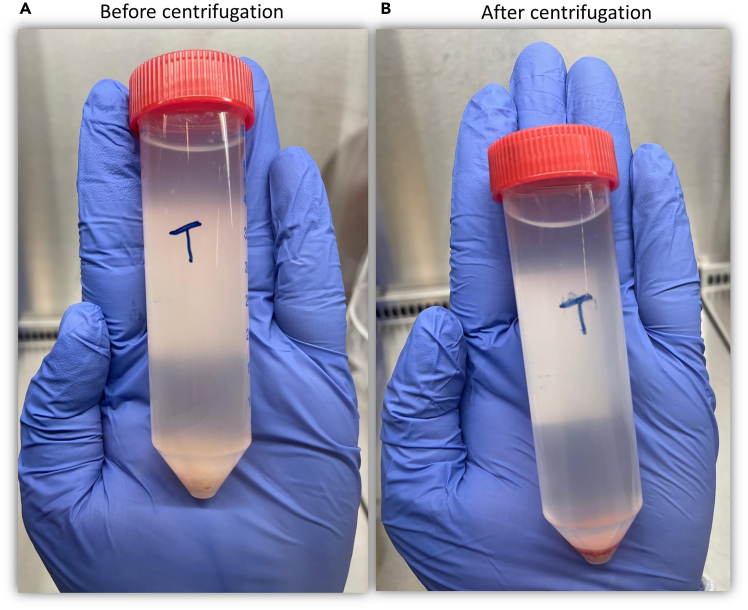
Image showing tube with minced before and after tissue grinding step 3 (A) An example of a minced tissue in DPBS showing the minced tissue at the bottom, (B) Depiction of the resulting tissue pellet mixed with red blood cells (RBCs), giving it a red appearance.

4. Add sufficient RPMI-1640 medium to adjust the total volume to 50 mL, then centrifuge at 500 × *g* relative centrifugal force (RCF) for 10 mins at 4°C (Figure 2).
5. Discard the supernatant and re-suspend the tissue with RPMI-1640 media.
  a. For mechanical digestion: For every 500 mg of minced tissue, add 800 μL RPMI-1640 to resuspend.
  b. For enzymatic digestion: Re-suspend the minced tissue pellet in 7.85 mL RPMI-1640 media (without FCS) for every 500 mg of tissue.

***Note:*** For instance, if the control tissue weighs 4.6 g, prepare nine 15 mL tubes. Transfer 7. 85 mL of the RPMI-1640-supplemented minced tissue suspension from each section into the appropriately labeled 15 mL tubes.

***Note:*** Calculate the total volume of RPMI-1640 media based on the tissue weight.

### Tissue dissociation

**Timing: 32.5 min per 2.0 g of tissue (for steps 6–15)**

**Timing: 125 min for 1 g of tissue (for steps 16–21)**

**Part 1: Mechanical digestion (refer to steps 6–15):** This section details the mechanical dissociation of minced liver tissue using the TissueGrinder. Prepare the TissueGrinder instrument and load 800 μL of the minced tissue suspension into the labeled TG tubes. Assemble the rotor unit, attach the TG tube with a 70 μm strainer, and place the setup onto the device. Start the dissociation run using the appropriate tumor tissue protocol in the software. When the LEDs turn green, remove the tube, invert it, and centrifuge. Open the tube carefully, wash the strainer with PBS, resuspend the pellet, and centrifuge again before discarding the supernatant.

**Figure 3:**
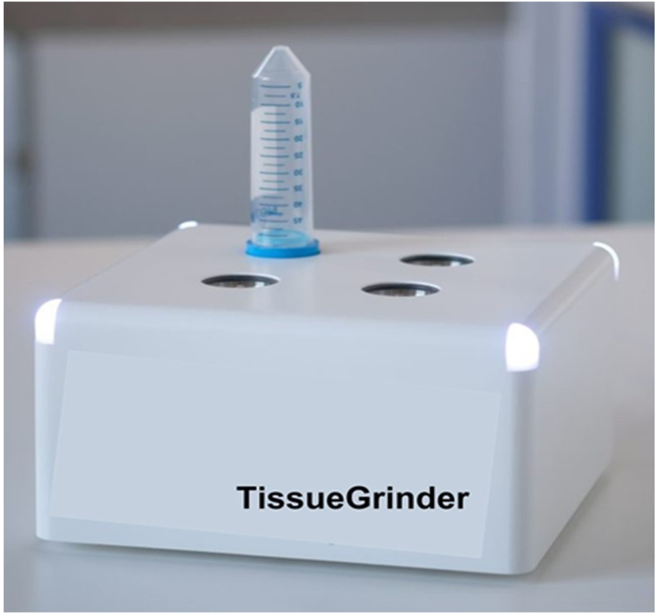
The figure illustrates the TissueGrinder instrument equipped with a TG tube (Created and modified in https://BioRender.com)

**Figure 4:**
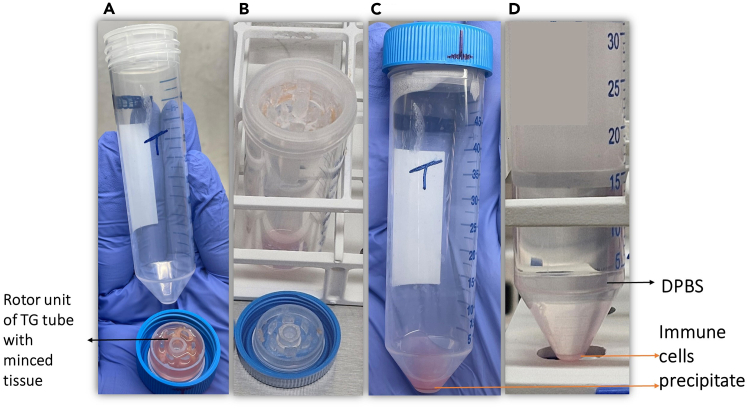
TG tubes before and after the grinding process (A) TG tube components, including the rotor unit, loaded with minced tissue in RPMI-1640 medium, (B) Top view of the TG tube, (C) Front view of the TG tube, (D) Front view of the TG tube.

**Figure 5:**
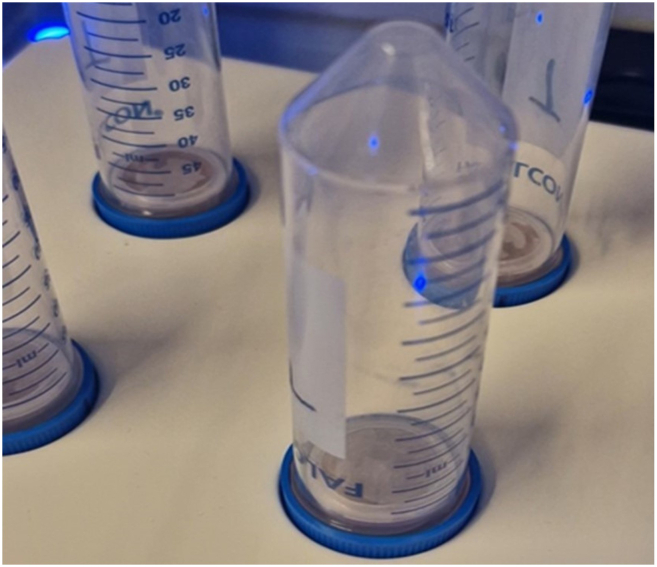
An example of showing how to insert TG tubes in TissueGrinder

6. Prepare the TissueGrinder instrument (Figure 3) (check preparation of instruments section).
7. As prepared in step 5a, transfer 800 μL of the RPMI-1640–supplemented 500 mg minced tissue suspension from both the tumor and non-tumor sections into the rotor units of the appropriately labeled TG tubes (Figure 4A).
8. Position the rotor unit in the lid of a 50 mL TG tube and place the TG tube with a 70 μm cell strainer on top of the rotor unit.
9. Place the 50 mL TG tube on the lid holding rotor unit containing minced tissue, screw it on, and position it on the TissueGrinder device (Figure 5).

***Note:*** Insert and align the tube in an upright position without tilting.

10. Open the TissueGrinder operating software, label the sample, and select tumor tissue protocol for non-tumor and tumor tissue section and press start to commence the dissociation process.

***Note:*** Watch the Methods Video S1 to observe the rotor motion within the TG tubes on the TissueGrinder during the dissociation step.

***Note:*** Follow the manufacturer’s grinding parameters and protocol, and refer to Table S1 for detailed procedures.

11. Upon completion of the dissociation protocol, check the LED indicators at the four corners of the instrument; they will change from blue to green to signal that the process is finished.
12. After the grinding procedure, invert the tube and centrifuge at 500 × *g* for 10 mins at 4°C (Figures 4B and 4C).
13. Place the TG tubes onto a rack and carefully open them without discarding any cells or supernatant.
14. Wash the cell strainer with 5 mL of PBS, and then resuspend the cell pellet.
15. Centrifuge the suspension at 500 × *g* for 10 mins at 4°C (Figure 4D). After centrifugation, discard the supernatant.

Methods Video S1. The video demonstrates the grinding motion of the rotor unit within the TG tubes mounted on the TissueGrinder instrument, related to Part 1 Mechanical digestion Tissue dissociation)

**Part 2: Enzymatic digestion (refer to step 16–21):** This section details enzymatic dissociation of liver tissue using Collagenase IV and DNase I. Add the enzyme solutions to the minced tissue, adjusting volumes to tissue weight. Mix thoroughly and incubate at 37°C on an orbital shaker with continuous agitation to promote digestion. After incubation, centrifuge, discard the supernatant, and add C-RPMI to stop the enzymes. Combine paired tubes, filter the suspension through a 40 μm strainer into a 50 mL tube, and rinse to maximize recovery. Wash the cells twice with RPMI-1640, centrifuging after each wash and removing the supernatant carefully.

**Figure 6:**
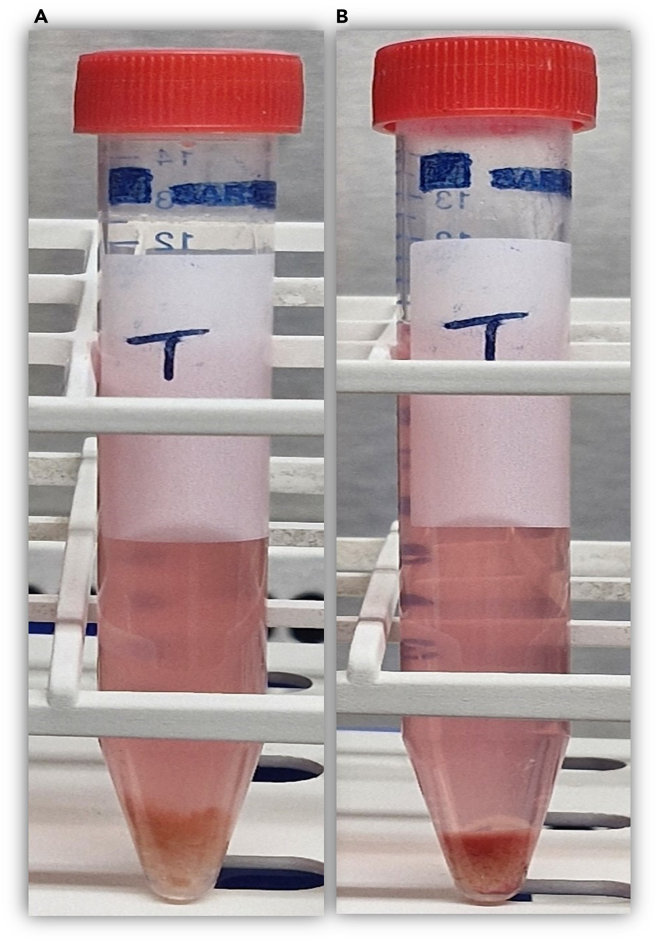
Illustration of liver tissue homogenate before and after enzymatic digestion (A) Example of tubes containing minced tissue in a mixture of RPMI-1640 medium with Collagenase IV and DNase I reagents, (B) Tube showing digested tissue in the enzymatic solution.

16. As prepared in step 5b, add 2 mL of Collagenase IV solution (1 mg/mL) and 150 μL of DNase I solution (1 mg/mL) to each tumor and non-tumor tubes (Figure 6A).

***Note:*** During tissue processing, keep the liver tissue at 4^o^C, ensuring it stays submerged in MACS tissue storage solution.

**CRITICAL:** Ensure that the weight of tissue transferred into each 15 mL tube and the total volume of the tissue suspension are accurately adjusted, as the concentrations of Collagenase IV and DNase I solutions are standardized per 0.5 g of tissue and per total suspension volume. Thoroughly mix the minced tissue suspension and aliquot the precise volume into each tube accordingly.

17. Place each tube on a temperature-controlled orbital shaker at 37°C with continuous agitation at 180 revolutions per minute for 60 mins.

***Note:*** The temperature and continuous agitation promote effective enzymatic interaction with the tissue suspension.

18. After revolutions, centrifuge the 15 mL test tubes at 429 × *g* for 10 mins at 24°C (Figure 6B).
19. Discard the supernatant and add 10 mL of C-RPMI to block Collagenase IV activity in each tube.
20. Combine the cell suspensions from two 15 mL tubes into a single 50 mL tube. Using a pipette, filter the cells through a 40 μm cell strainer into a new 50 mL tube. Rinse the strainer by adding 10 mL of RPMI-1640 medium to ensure maximum cell recovery.
21. Wash the cells twice with 20 mL of RPMI-1640 medium. Centrifuge at 429 × *g* for 10 mins at 4°C and carefully decant the supernatant after each spin.

### Immune cell isolation

**Timing: Processing 4.0 ± 1 g of tissue to isolate lymphocytes takes approximately 70 min**

For mechanical digestion, refer to steps 22, and 24–33; for enzymatic digestion, refer to steps 23 and 24–33. This section describes isolating primary immune cells from mechanically or enzymatically dissociated liver tissue. Add 33% Percoll to the cell pellet, resuspend in a clean 50 mL tube, and centrifuge under low-brake conditions. Aspirate the supernatant without disturbing the loose immune cell pellet, lyse red blood cells, and keep tumor and non-tumor samples separate. Wash the cells twice with cold PBS, centrifuging between washes, then resuspend in FACS buffer. Filter through a 40 μm strainer, assess viability and cell counts with Trypan blue and an automated counter, and keep the isolated immune cells at 4°C until flow cytometry.

**Figure 7:**
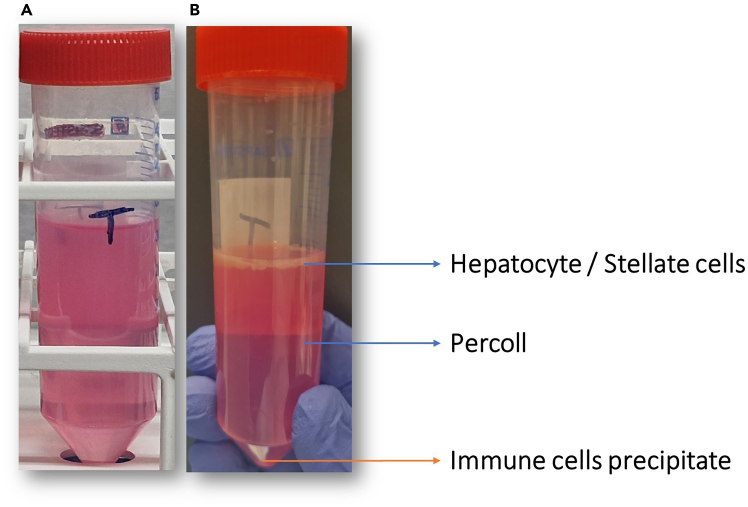
Setup of the Percoll gradient (A) Example of a prepared gradient before centrifugation, (B) Illustration of the gradient layers formed after centrifugation, with the desired immune cell pellet collected by discarding the top fractions.

22. For mechanical digestion, proceed with the steps outlined below.
  a. Add 30 mL of 33% Percoll stock solution directly to the TG tube containing the cell pellet from tumor and non-tumor tissue sections.
  b. Transfer cell suspension from TG tube into new 50ml tube due to differences in weight and non-sealing strainer lid of TG tubes.
  c. Resuspend the pellet in the Percoll solution to ensure a uniform suspension before centrifugation (Figure 7A).
23. For enzymatic digestion: follow the steps below.
  a. Add 30 mL of 33% Percoll stock solution directly to the tube containing the cell pellet from tumor and non-tumor tissue sections.
  b. Briefly pipette and resuspend the cell pellet in the Percoll solution before centrifugation (Figure 7A).
24. Spin the 50 mL tubes at 1000 × *g* for 20 mins at 24°C, with acceleration set to 1 and brake set to 0 (Figure 7B).

***Note:*** Do not activate the brake, as it may disturb the separation gradient and reduce cell yield.

25. Aspirate the supernatant gently after centrifugation to avoid disrupting the immune cell pellet at the bottom of the 50 mL tubes.

**CRITICAL:** The cell pellet remains loosely suspended and does not firmly adhere to the bottom of the conical tube; therefore, carefully aspirate the supernatant to avoid unintentional loss of liver tissue–derived immune cells.

26. Resuspend the isolated primary immune cell pellet in 5 mL RBC lysis buffer and incubate for 5 mins at 20°C–25°C.
27. After incubation, add 10 mL of cold PBS. Pool the cells with RBC lysis buffer from all tumor sections into one or more 50 mL tubes, and pool those from all non-tumoral sections into separate 50 mL tubes.
28. Fill each tube of tumor and non-tumoral sections with cold 1× PBS to 50 mL, then centrifuge at 877 × *g* for 10 mins at 4°C.
29. Gently discard the supernatant and resuspend the pellet in 50 mL cold 1× PBS, then centrifuge at 877 × *g* for 10 mins at 4°C.
30. Gently discard the supernatant and resuspend the cell pellet in 1 mL FACS buffer.
31. Filter the cells from all tubes using a pipette through a 40 μm cell strainer into a 15 mL tube.
32. Mix 10 μL of the cell sample with 10 μL of Trypan blue at a 1:1 ratio. Load 10 μL of the dilution onto a Luna Cell Counter slide and count the cells using the pre-established protocol for mononuclear cells.
33. Keep the cells at 4°C until the flow cytometry downstream application.

### Flow cytometry staining

**Timing: Staining lymphocytes isolated from 4.0 ± 1 g of tissue takes approximately 70 min**

For mechanical digestion, refer to steps 34-47; for enzymatic digestion, refer to steps 34-47. This section describes staining mononuclear cells for flow cytometry. Prepare a fresh antibody master mix and keep it at 4°C. Pellet 0.5–2 × 10^6^ viable cells, wash with FACS buffer, and block Fc receptors before adding the antibody master mix. Incubate the cells on ice in the dark, wash, and stain for viability using Zombie NIR. Wash again, resuspend the final pellet in FACS buffer, and keep the samples at 4°C. Acquire data on a flow cytometer and analyze FCS files using FlowJo the same day.

34. Prepare antibody Master Mix for surface markers for tissue-isolated mononuclear cells (MNCs) as shown in Table 1.

**Table 1:** Antibody and dye staining master-mix, related to step 34

Dye		Volume (μL)
Zombie NIR Fixable Viability Kit (Reconstituted)		1
Human TruStain FcX		1

Antibody	Volume (μL)	Concentration (μg/100 μL)

CD45 APC/Fire810	1	0.1
Lineage CD34 FITC	1.6	0.16
Lineage CD123 FITC	1.6	0.32
Lineage CD14 FITC	1.6	0.64
Lineage CD19 FITC	1.6	0.64
Lineage CD11c FITC	1.6	0.64
Lineage FcεRIα FITC	1.6	0.32
Lineage TCRαβ FITC	1.6	0.64
Lineage TCRγδ FITC	1.6	0.96
CD3 BV570	1.6	0.13
CD4 BV650	1.6	0.16
CD8a BV510	1.6	0.16
CD16 AF700	1.6	0.79
CD56 BV605	1.6	0.16
CD94 PE/Dazzle 594	1.6	0.16
CD161 BV785	1.6	0.16
CD127 BV711	1.6	0.16
	Total volume of antibody master-mix =34.5	
	Total FACS buffer volume = 65.5	
	Total staining volume: (34.5 + 65.5 = 100 μL)	

**Storage conditions**: Prepare the master mix fresh, keep it at 4°C, and use it on the same day.

35. Place 0.5–2 × 10^6^ viable cells into FACS staining tubes, add FACS buffer to bring the volume to 3 mL, and centrifuge the tubes at 877 × *g* for 5 mins at 4°C.

***Note:*** Calculate the number of viable cells based on the cell count obtained in step 21.

36. Discard the supernatant, resuspend the cell pellet in 99 μL FACS buffer, and add 1 μL Human Tru Stain FcX. Pipet up and down to mix and incubate for 10 mins at 4°C.

***Note:*** Block Fc receptors by incubating the cells with Fc blocker to prevent non-specific antibody binding.

37. Centrifuge the tubes at 877 × *g* for 5 mins at 4°C.
38. Discard the supernatant, resuspend the cell pellet in 65.5 μL FACS buffer, and add 34.5 μL fluorochrome-conjugated antibodies master mix in each sample prepared in the step 16. Pipet up and down to mix.
39. Incubate cells with Antibody Master Mix for 20 mins at 4°C in dark.

**CRITICAL:** Make sure to protect the cell suspensions from light exposure.

40. Following incubation, add FACS buffer to bring the volume to 3 mL, and centrifuge the tubes at 877 × *g* for 5 mins at 4°C.
41. Resuspend the cell pellet in 99 μL ice cold PBS, and add 1μL Zombie NIR dye and incubate cells for 10 mins at 4°C.
42. Following incubation, add 3 mL cold FACS buffer.
43. Centrifuge the tubes at 877 × *g* for 5 min at 4°C.
44. Gently aspirate supernatant without disrupting the cell pellet.
45. Add 500 μL FACS buffer and resuspend the cell pellet by pipetting up and down. Store at 4°C.
46. Acquire the data using available Flow Cytometer.
47. Analyze Flow Cytometry Standard (FCS) files using FlowJo analysis software.

***Note:*** Fluorescently labeled cells should be analyzed by flow cytometry on the same day to ensure accurate final readout.

## Expected outcomes

Both enzymatic and mechanical (TissueGrinder-based) tissue digestion methods yielded comparable cell numbers and showed high lymphocyte viability. However, enzymatic digestion using Collagenase IV and DNase I resulted in higher contamination with hepatocytes, stellate cells, and other residual cell types, whereas the mechanical method produced a purer lymphocyte population. From ∼1 g liver tissue, a yield of ∼2 × 10^6^ cells from tumor and ∼1 × 10^6^ cells from non-tumor tissue, with viability above 85% should be expected when counting directly after isolation. The enzymatic method resulted in ∼50% residual cells in the non-tumor section and ∼35% in the tumor section, whereas the mechanical method resulted in ∼35% in the non-tumor section and ∼30% in the tumor section. These findings indicate that mechanical digestion enables more efficient isolation of mononuclear cells.

In terms of time efficiency, the enzymatic approach required approximately 115 minutes per 0.5 grams of tissue, while the TissueGrinder method completed digestion in only 22.5 minutes for the same amount of tissue. Cost analysis based on 2024 reagent prices indicated that both approaches are broadly comparable, although the enzymatic method was less economical (≈€ 24.6 per 0.5 g) than the mechanical approach (≈€ 10.5 per 0.5 g). Both techniques demonstrated consistent reproducibility across different tissue morphologies. Notably, the cost comparison focused on the most distinctive reagents—Collagenase IV and DNase I for the enzymatic method and TissueGrinder (TG) tubes for the mechanical method.

Mechanical digestion could be chosen for faster, more economical, and gentle tissue processing. Selection of the tissue digestion method based on several factors, as enzymatic treatments can alter or degrade cell surface proteins and damage fragile or sensitive immune cell populations isolated from tissue. The choice of digestion approach depends on the specific downstream applications and functional requirements of the isolated immune cells. Both protocols eliminate the need for Percoll gradient layering, which is time-consuming and prone to errors such as inaccurate layering, gradient mixing from shaking, or miscalculated concentrations.

**Figure 8:**
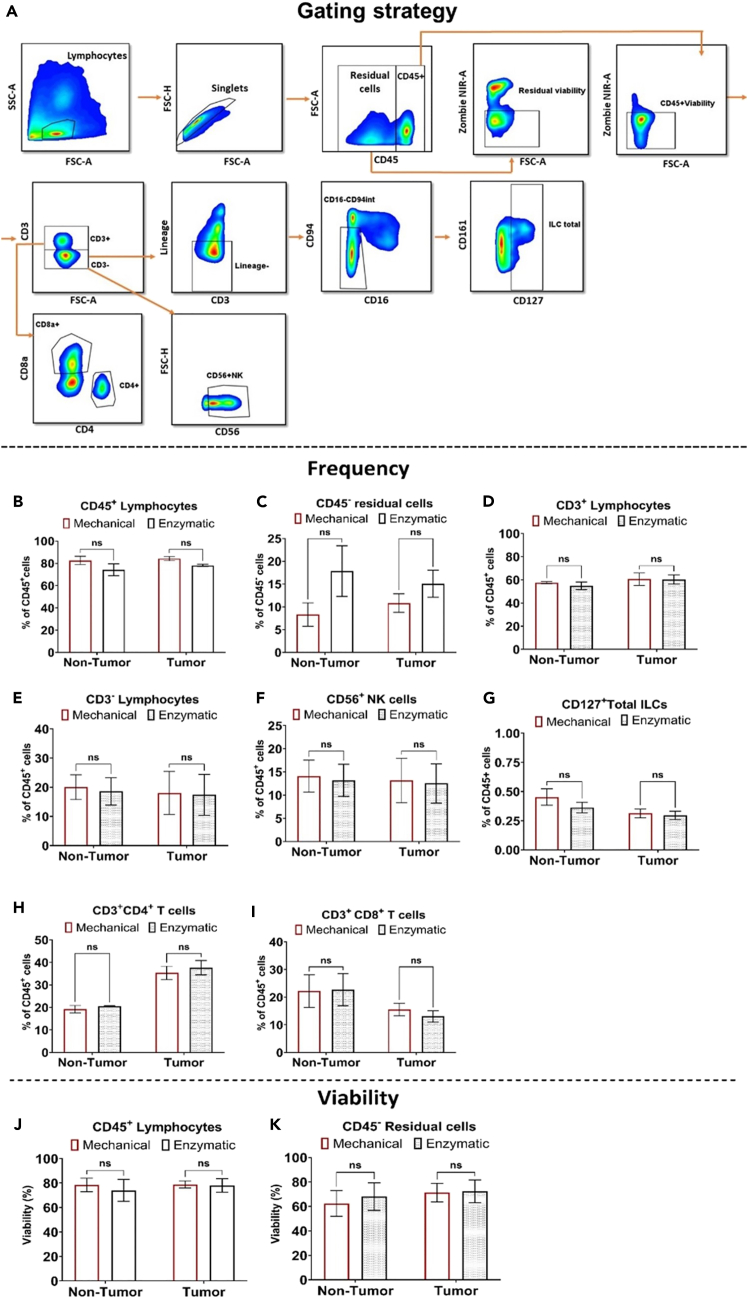
Analysis of MNCs isolated from non-tumor and tumor liver tissue using mechanical and enzymatic methods Human liver non-tumor (n = 4) and tumor (n = 4) tissue were processed to isolate MNCs, followed by flow cytometry staining and analysis. (A) Flow cytometry gating strategy: The composition of single-cell suspensions and lymphocytes isolated from tissue biopsies was analyzed. Doublets and dead cells were excluded. The frequencies of lymphocytes (CD45^+^), NK cells (CD45^+^CD3^-^CD56^+^), and ILCs (CD45^+^CD3^-^Lineage^-^CD16^-^CD95^int^CD161^+^CD127^+^) are presented as dot plots, (B–I) Comparison of isolation methods: The mechanical and enzymatic methods were compared to assess differences in the frequency of MNCs, including lymphocytes, residual cells, NK cells, total ILCs, and T cell subsets, (J and K) Viability Assessment: The viability of CD45^+^ lymphocytes and CD45^-^ residual cells was compared between the mechanical and enzymatic methods. The results were analyze with Multiple Wilcoxon tests, ns (0.12), ∗p value <0.0332, ∗∗p value <0.0021, ∗∗∗p value <0.0002, ∗∗∗∗p value <0.0001.

The liver can be recognized as a metabolic organ that reflects an individual’s health and consists of diverse cell types, including hepatocytes, endothelial cells, Kupffer cells, stellate cells, and others.^9^ When HCC develops, the liver undergoes morphological changes and increased infiltration of immune cells targeting cancer cells.^10^^,^^11^ To preserve the native phenotype and functionality of rare immune cell populations within the tumor and the tumor microenvironment, it is essential to employ a tissue dissociation method that minimizes cellular stress and preserves molecular and genetic integrity. In this study, a customized flow cytometry panel and gating strategy were developed and applied to accurately identify and characterize rare immune cell subsets, including CD127^+^ ILCs and CD56^+^ NK cells (Figure 8A).

Flow cytometry reveals no statistical significant differences (“ns”) in the frequency of major immune populations or T cell subsets between mechanical and enzymatic digestion across tumor and non-tumor tissues (Figures 8B–8I). In tumor samples, mechanical digestion yields 6.12% more immune cells and 4.25% fewer residual cells than enzymatic digestion. It also produces slightly higher frequencies of CD3^+^, CD3^−^, CD4^+^, CD8^+^, and NK cells, demonstrating an advantage in cell recovery and immune subset representation.

Viability remains high and comparable for tumor-infiltrating cells, while mechanical digestion improves non-tumor immune cell viability by 4.5%. Both methods preserve the viability of residual cells equally (Figures 8J and 8K). Overall, mechanical digestion consistently matches enzymatic digestion in preserving cell frequency and viability, while avoiding enzyme-induced damage. It offers a simple, enzyme-free, and reliable approach for isolating viable immune cells from liver tissues, ideal for immune profiling of the HCC microenvironment.

Followed by frequency, the absolute cell counts were calculated based on the frequency of cells obtained from flow cytometry frequency data and the manual cell count and shown in the formula below:

AbsoluteCellNumber=(FrequencyofCellPopulation(%)/100)×TotalCellCountfromManualCount

**Figure 9:**
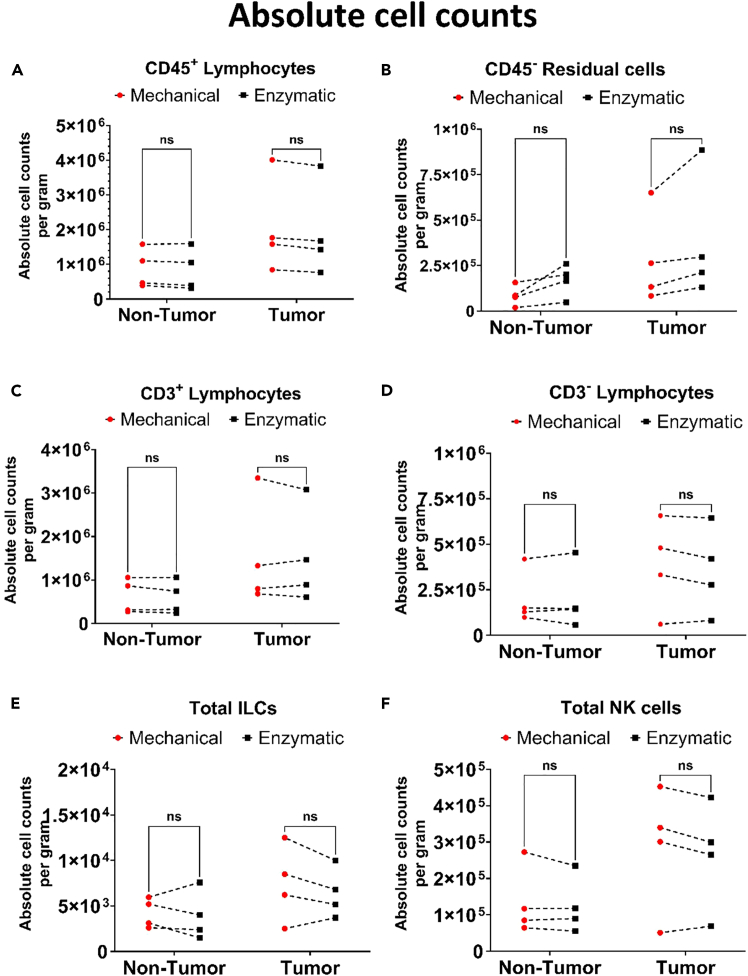
Analysis of absolute cell counts per grams of MNCs isolated from non-tumor and tumor liver tissue using mechanical and enzymatic methods Human liver non-tumor (n = 4) and tumor (n = 4) tissue were processed to isolate MNCs, absolute cell counts were calculated based on the frequency and cell counts, (A–F) The absolute cell counts were calculated and compared between mechanical and enzymatic method for lymphocytes, residual cells, CD3^+/−^ lymphocytes, Total ILCs, Total NK cells. The results were analyze with Multiple Wilcoxon tests, ns (0.12), ∗p value <0.0332, ∗∗p value <0.0021, ∗∗∗p value <0.0002, ∗∗∗∗p value <0.0001.

Flow cytometry shows no significant differences in absolute immune cell counts between mechanical and enzymatic digestion across both tumor and non-tumor tissues (Figure 9). Mechanical digestion consistently matches enzymatic yields while eliminating the need for enzyme treatment that may damage surface markers or compromise cell viability. In tumor samples, mechanical digestion produces a 1.06-fold higher yield of CD45^+^ lymphocytes and a 0.74-fold lower yield of residual cells compared with enzymatic digestion (Figures 9A and 9B). Although CD3^+^ and CD3^−^ lymphocyte numbers remain comparable between methods (Figures 9C and 9D), mechanical digestion achieves a 1.15-fold increase in low-frequency ILC recovery (Figure 9E). This enrichment of ILCs is particularly important, as these rare subsets often escape detection in enzyme-based protocols, limiting downstream immune profiling. By enhancing recovery of ILCs without compromising viability, mechanical digestion provides a clear advantage for studies that require precise analysis of low-frequency immune populations.

Additionally, the number of NK cells isolated using mechanical digestion was approximately 1.08-fold higher than with enzymatic digestion (Figure 9F). These findings support the use of the mechanical digestion method as a reliable and robust approach for isolating immune cells from liver tissues, while avoiding potential artifacts introduced by enzymatic digestion. Furthermore, mechanical digestion offers a gentler alternative that preserves cellular integrity and surface marker expression, which is crucial for downstream applications such as flow cytometry and functional assays.

**Figure 10:**
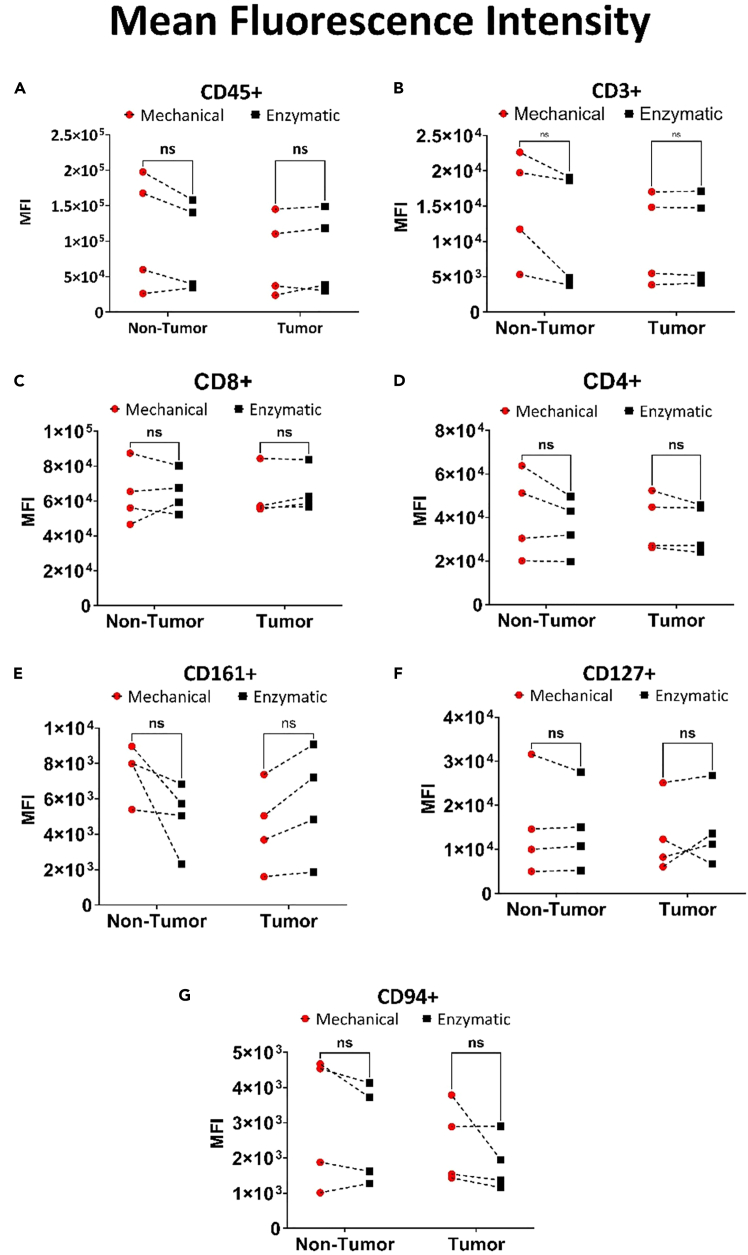
Fluorescence intensity shift in cell populations due to stress induced by mechanical and enzymatic processing methods Human liver non-tumor (n = 4) and tumor (n = 4) tissue were processed to isolate MNCs, followed by flow cytometry staining with specific antibodies. MFI values were obtained using FlowJo analysis, (A–G) Comparison of MFI Values: The MFI values for CD45, CD3, CD8, CD4, CD161, CD127, and CD94 were compared between mechanical and enzymatic methods. Statistical analysis was performed using multiple Wilcoxon tests, with significance levels indicated as follows: ns (not significant, p > 0.12), ∗p < 0.0332, ∗∗p < 0.0021, ∗∗∗p < 0.0002, ∗∗∗∗p < 0.0001.

We evaluated the impact of mechanical and enzymatic digestion on isolated immune cells by measuring cell surface marker expression through MFI. We compared both methods to determine whether tissue processing altered marker stability or compromised phenotypic integrity, and we used these findings to judge the reliability of each approach for downstream immune profiling. Mechanical digestion provided slightly higher MFI as equivalent of higher expression levels of several analyzed cell surface markers, although not statistically significant when compared to enzymatic method (Figure 10). In tumor samples, mechanical digestion results in a higher MFI for CD45 lymphocytes, showing a 19,679 increase compared to enzymatic digestion (Figure 10A). When examining non-tumor samples, mechanical digestion also yields a 3,255 higher MFI for CD3^+^ immune cells (Figure 10B). Furthermore, CD161 MFI is 2,612 higher in mechanically digested samples than in enzymatically digested ones (Figure 10E), while receptors like CD8, CD4, CD127, and CD94 show no significant difference across methods (Figures 10C, 10D, 10F, and 10G). Mechanical digestion maintains high viability comparable to enzymatic digestion for tumor-infiltrating cells, while improving the viability of non-tumor immune cells by 4.5%. Overall, it preserves cell frequency and viability as effectively as enzymatic digestion, but without enzyme-induced damage or unwanted alterations of cell surface markers.

## Limitations

The protocol described here has proven effective, but it comes with some limitations that researchers should consider. We have tested this protocol using the TissueGrinder technology for tissue digestion, but alternative devices like the GentleMACS Tissue Dissociator, while possibly applicable, have not been tested in our hands. One key limitation of mechanical grinding using scalpel is that the force and duration of grinding may vary, leading to inconsistent results. If too much pressure or time is applied, the tissue may be over-digested, while insufficient pressure or time may result in incomplete dissociation. Additionally, adding cell dissociation enzymes during mechanical digestion could further reduce residual cell. This combined approach has not been tested in our protocols, and its effectiveness remains uncertain. Another consideration is that liver biopsies were not perfused in our protocol, which may lead to partial contamination from peripheral blood immune cells. Finally, residual liver cells may exhibit autofluorescence, which can complicate the discrimination of various immune cell types during flow cytometry analysis. To overcome this, it is essential to include appropriate controls for accurate gating during FACS analysis. Despite these challenges, the protocol described enables efficient isolation of mononuclear cells for downstream applications.

## Troubleshooting

### Problem 1

**Low cell yield:** Tumor morphology influences mononuclear cell yield by affecting tissue dissociation efficiency and cell quality. (a) Dense or fibrotic tumors are harder to dissociate into single-cell suspensions, (b) Poorly vascularized tumors may have fewer infiltrating immune cells, resulting in lower yields, and (c) Tumors with necrosis or hypoxia often show reduced viability and altered cell composition.

**CRITICAL:** During Tissue dissociation (tumor processing stage) [Refer to: Tissue grinding]

### Potential solution

To improve mononuclear cell yield from tumor tissues, optimize dissociation protocols using efficient enzymes or mechanical devices, and pre-treat dense or fibrotic tumors for easier breakdown. Adjust incubation conditions for better cell recovery. Optimize centrifugation parameters for improved separation. These strategies can help maximize both the yield and quality of isolated mononuclear cells for downstream analyses.

### Problem 2

**Low cell viability:** Prolonged exposure to collagenase digestion, the red blood cell lysis step, and/or high rotor speeds during Collagenase IV incubation lowers cell viability.

**CRITICAL:** During Enzymatic tissue digestion in Part 2 and red blood lysis following density gradient centrifugation in both Part 1 and Part 2 methods [Refer to : Tissue dissociation.

### Potential solution

Minimize minced tissue suspension exposure to collagenase to preserve cell viability. Control the revolutions per minutes of the orbital platform during enzymatic digestion to avoid excessive shear forces, which may damage cells. Limit the duration of the red blood cell lysis step to reduce stress on the cells and preserve their viability. After one round of RBC lysis, if red blood cells remain in the suspension, repeat the lysis step without extending incubation beyond the recommended time.

### Problem 3

**Contamination in final yield:** When many cells die and release DNA into the suspension, several issues can arise: (a) increase of viscosity, (b) reduced cell yield and quality, (c) cell death cascade.

**CRITICAL:** During tissue dissociation and immediate post-dissociation handling (including RBC lysis and pre-FACS preparation) [Refer to: Tissue dissociation]

### Potential solution

Control dissociation conditions carefully to avoid these issues. Minimize exposure time to damaging enzymes or mechanical forces. Incorporate appropriate washing and filtering steps to remove free DNA from the suspension. Use DNase I in FACS buffer to break down free DNA and prevent it from interfering with your experiments.

### Problem 4

**Clumping of cells:** Clumping of cells occurs during the isolation process due to dead cells and cell debris.

**CRITICAL:** During post-isolation handling and pre-flow cytometry preparation [Refer to: Tissue dissociation].

### Potential solution

To prevent this, optimize each step carefully for tissues with distinct morphologies. After isolating cells from the tissue and before flow cytometry acquisition, add an additional step to strain the cell suspension using 40 μm filters to remove clumps. Re-suspend the cells in a higher volume of FACS buffer to avoid clogging the flow cytometer.

### Problem 5

**Autofluorescence:** The presence of residual or non-immune cells, such as epithelial cells and hepatocytes, contributes to autofluorescence.

**CRITICAL:** During post-dissociation suspension preparation and flow cytometry acquisition [Refer to: Tissue dissociation].

### Potential solution

Residual and non-immune cells exhibit significant autofluorescence. Therefore, include appropriate controls, such as unstained healthy donor-derived MNCs or cell suspension from tissue digestion, to confirm that the positive signal is valid.

### Problem 6

**The structural organization of the tissue**, including factors such as density, fibrous content, and extracellular matrix composition, can interfere with the grinding process by making the tissue more resistant to mechanical disruption and leading to incomplete dissociation.

**CRITICAL:** During mechanical dissociation with the TissueGrinder (tissue processing stage) [Refer to: Tissue grinding].

### Potential solution

Tissue structural changes, such as fibrosis, can transform the liver from a soft, organized, and vascular tissue into a stiff, scarred organ with distorted architecture, regenerative nodules, and impaired blood flow. In such cases, additional grinding may be required to reduce the tissue size sufficiently for proper placement inside the rotor unit of the TG tubes. Apart from this adjustment, the instrument includes a protocol specifically optimized for tumor tissues, which also accommodates the challenges associated with fibrotic and necrotic samples.

## Resource availability

### Lead contact

Further information and requests for resources and reagents information should be directed to and will be fulfilled by the lead contact, Dr. med. Bernd Heinrich (heinrich.bernd@mh-hannover.de).

### Technical contact

Further information and request regarding protocol, procedure, and troubleshooting should be directed to and will be fulfilled by the technical contact, Dr. Sachin Kumar Singh Chauhan (chauhan.sachin@mh-hannover.de).

### Materials availability

No new material was produced using this protocol.

### Data and code availability

The published article includes all datasets generated or analyzed during this study.

## Acknowledgments

B.H. receives funding from the Max Eder junior research group program of the German Cancer Aid. S.K.S.C. received funding via “Förderstiftung MHHplus.” We would like to thank Dr. Alexander Kroemer (MedStar Georgetown Transplant Institute, Washington, DC, USA) who provided the initial protocol for enzymatic digestion as well as Dr. Firouzeh Korgany and Dr. Tim Greten (both National Institutes of Health, Bethesda, MD 20892, USA) for providing resources to refine the protocol to study innate lymphoid cells as referenced here.^2^^,^^12^ We would specifically like to thank Stefan Scheuermann, Eveline Priebe, and Felix Dirla from Fast Forward Discoveries for providing the TissueGrinder setup and associated training and fruitful discussions.

## Author contributions

S.K.S.C.: methodology, study designing, conceptualization, writing – original draft, and writing – review and editing; L.F.: supervision of surgeries and sample curation; M.S.: supervision and providing infrastructure; H.W.: supervision and providing infrastructure; B.H.: reviewing, editing, and supervision.

## Declaration of interests

The authors declare no competing interests.

## Declaration of generative AI and AI-assisted technologies in the writing process

During the preparation of this work, the author used OpenAI (2025)-*ChatGPT* (*GPT-5*) (large language model): https://chat.openai.com/ in order to check English grammar. After using the tool, the authors reviewed and edited the content as needed and take full responsibility for the content of the publication.

## Supplemental information

Table S1. Tumor tissue protocol applied during mechanical digestion, related to major step 10
