# Supplementary material for: Protocol for optimized mononuclear cell isolation from liver and tumor tissue using mechanical or enzymatic digestion
Source: STAR Protoc. 2025 Dec 19;7(1):104289. doi: 10.1016/j.xpro.2025.104289 (PMC12775951; doi:10.1016/j.xpro.2025.104289)
Supplement: Table S1. Tumor tissue protocol applied during mechanical digestion, related to major step 10 [file mmc1.pdf]

Table S1: Tumor tissue protocol applied during mechanical digestion, related to major step 10.

Protocol Name: Tumor tissue

Step 1

Rpm: -10

Duration: 00:00:20

Acceleration Time: 00:00:05

Step 2

Rpm: 15

Duration: 00:00:20

Acceleration Time": 00:00:05

Step 3

Rpm: -20

Duration: 00:00:20

Acceleration Time: 00:00:05

Step 4

Rpm: -25

Duration: 00:00:25

Acceleration Time: 00:00:05

Step 5

Rpm: 25

Duration: 00:00:25

Acceleration Time: 00:00:05

Step 6

Rpm: -15

Duration: 00:00:25

Acceleration Time: 00:00:05
